# Origami-inspired folding assembly of dielectric elastomers for programmable soft robots

**DOI:** 10.1038/s41378-022-00363-5

**Published:** 2022-03-31

**Authors:** Yanhua Sun, Dengfeng Li, Mengge Wu, Yale Yang, Jingyou Su, Tszhung Wong, Kangming Xu, Ying Li, Lu Li, Xinge Yu, Junsheng Yu

**Affiliations:** 1 State Key Laboratory of Electronic Thin Films and Integrated Devices, School of Optoelectronic Science and Engineering, University of Electronic Science and Technology of China (UESTC), Chengdu, 610054 People’s Republic of China; 2 Chongqing Key Laboratory of Materials Surface & Interface Science, Chongqing Co-Innovation Center for Micro/Nano Optoelectronic Materials and Devices, Micro/Nano Optoelectronic Materials and Devices International Science and Technology Cooperation Base of China, School of Materials Science and Engineering, Chongqing University of Arts and Sciences, Chongqing, 402160 People’s Republic of China; 3 Department of Biomedical Engineering, City University of Hong Kong, Hong Kong SAR, 999077 People’s Republic of China

**Keywords:** Electrical and electronic engineering, Electronic properties and materials

## Abstract

Origami has become an optimal methodological choice for creating complex three-dimensional (3D) structures and soft robots. The simple and low-cost origami-inspired folding assembly provides a new method for developing 3D soft robots, which is ideal for future intelligent robotic systems. Here, we present a series of materials, structural designs, and fabrication methods for developing independent, electrically controlled origami 3D soft robots for walking and soft manipulators. The 3D soft robots are based on soft actuators, which are multilayer structures with a dielectric elastomer (DE) film as the deformation layer and a laser-cut PET film as the supporting flexible frame. The triangular and rectangular design of the soft actuators allows them to be easily assembled into crawling soft robots and pyramidal- and square-shaped 3D structures. The crawling robot exhibits very stable crawling behaviors and can carry loads while walking. Inspired by origami folding, the pyramidal and square-shaped 3D soft robots exhibit programmable out-of-plane deformations and easy switching between two-dimensional (2D) and 3D structures. The electrically controllable origami deformation allows the 3D soft robots to be used as soft manipulators for grasping and precisely locking 3D objects. This work proves that origami-inspired fold-based assembly of DE actuators is a good reference for the development of soft actuators and future intelligent multifunctional soft robots.

## Introduction

Soft robots have irreplaceable advantages in mechanical and biomedical engineering^1–4^. Because of their soft bodies, soft robots can adapt their body shape to complex physical environments and walk through narrow passages that rigid robots cannot^5,6^. The soft nature of their bodies also prevents sharp injuries to objects they touch, allowing them to enter the human body for drug transport^7^ or act as an operator in medical robotic systems during clinical surgeries^8–10^. In addition, soft actuators have been widely used as manipulators in large demission robots^11^. In practice, to increase their maneuverability for robotic walking, medical manipulation, and three-dimensional (3D) object grasping, it is crucial to build soft robots with flexible and variable 3D structures.

Currently, 3D soft robots are typically manufactured through 3D printing and assembly with small actuators^12,13^. Compared to 3D shapes, 2D shapes are more space-efficient in terms of their spatial dimension^14^. Thus, origami-inspired 3D soft robot construction, derived from an inherent simplified and low-cost folding-based assembly technique, is a good strategy due to its ability to perform out-of-plane deformations for 3D structure construction and to switch between 2D and 3D^15–19^. The detailed advantages of origami robots are as follows: (i) Soft, simple preparation process, and low cost. Compared with traditional robots, origami robots have a more streamlined structure, eliminating many complex transmission gear structures and requiring less production material. (ii) High degree of collapse and space efficiency. Origami robots have less transportation and storage requirements since they are capable of converting from two- to three-dimensional shapes. (iii) Scalability and various applications. The diversity of the origami method enables robots to be highly scalable in terms of structure and functionality. However, more novel designs and research on origami robots are urgently needed to achieve complex functionality.

Scientists have developed different types of origami-inspired soft robots with a variety of materials and actuation methods, each with specific mechanical manipulation functions and movement styles^20^. For instance, a battery-free miniature origami robotic arm based on origami actuation was developed with shape memory alloys and has been used for arm orientation control and object grasping^21^. 2D nanomaterials, such as MXene^22^ and graphene^23,24^, have been used as functional layers in soft actuators and robots, with light fields typically used as an actuation method for 3D structural control, programmable actuation, movements, and various artistic displays. A new triple-layer dual-chip actuator based on photothermal actuation was successfully used to assemble a fast crawling soft robot and a powerful mechanical clamp^25^. In addition, 3D structures fabricated by the kirigami technique in phase-change liquid crystal elastomers are a new type of robotic technology, with light beams serving as the actuation source to control motion and steer the movement direction in 2D^26^. For the active folding assembly to interchange between 2D and 3D and repeatably deform, the actuator must be a soft deformation material. In addition to the materials mentioned above, dielectric elastomers are an excellent choice due to their relatively large actuation force with large deformations^27,28^. Moreover, dielectric elastomers are actuated by electrical energy, which is convenient for future integration with robotic systems.

Soft robots should be developed with intelligent sensing systems. A class of actuators that combine tensile and torsional deformation to achieve sensing and various motions has been investigated^29^. Humidity-driven fiber muscles detect changes in external humidity while twisting and stretching^30^. Twisted elastomeric fibers fitted with carbon nanotube sheaths and contact clasps for sensing can monitor resistance signals during electrothermally driven twisting^31^. In addition, a spiral fiber crawling robot, which simulates the musculoskeletal structure of a human arm, can detect body deformations while crawling with resistive strain sensors^32^. These findings have been used to develop intelligent textiles and soft robots that can perceive, interact with and adapt to environmental stimuli. Future intelligent robotic systems will inevitably be controlled automatically^33,34^. When combined with intelligent sensing systems^35–38^, soft and durable robotic systems can assist humans with long-term tasks through human–machine interactions^39–43^. Electrical actuation allows robots to be precisely controlled in various environments as long as driving programs are established. At present, nearly all functional intelligent robots are electrically driven due to the advantages of electrical actuation in terms of handling precision. In recent years, an increasing number of soft robots have been powered by electrical energy based on electrothermal^44^, piezoelectric^45^, and dielectric^32^ principles. Programmable electrical actuation, such as independent leg control of multilegged robots^46^ and segmented control of single-body robots^47^, enables soft robots to move and function in various ways. In contrast, dielectric elastomers have rarely been combined with origami design for electrically actuated paper-folding robots. For the first time, we introduced VHB4910 elastomers into origami-inspired robots because of their outstanding advantages, such as their quick response time and superior resilience.

In this work, we combined origami technology and electronically controlled actuators to develop programmable 3D soft robots that can reversibly switch between 2D and 3D structures. These soft robots rely on a variety of programming controls to assemble multiple origami structures and to perform functions such as walking, grasping, and locking objects. These electrical actuators are composed of a pre-stretched dielectric elastomer with conductive carbon grease on both sides, which functions as a stretchable conductive electrode, and a laser-cut polyethylene terephthalate (PET) film, which functions as a flexible support frame. By designing the shape of the PET frame, a wide range of 3D origami assemblies can be produced in a cost-effective and easy-to-process manner. These origami-inspired soft robots based on electronically controlled dielectric elastomers perform well in terms of movement, assembly, and function, serving as good models for future 3D soft robot construction.

## Results and discussion

**Fig. 1:**
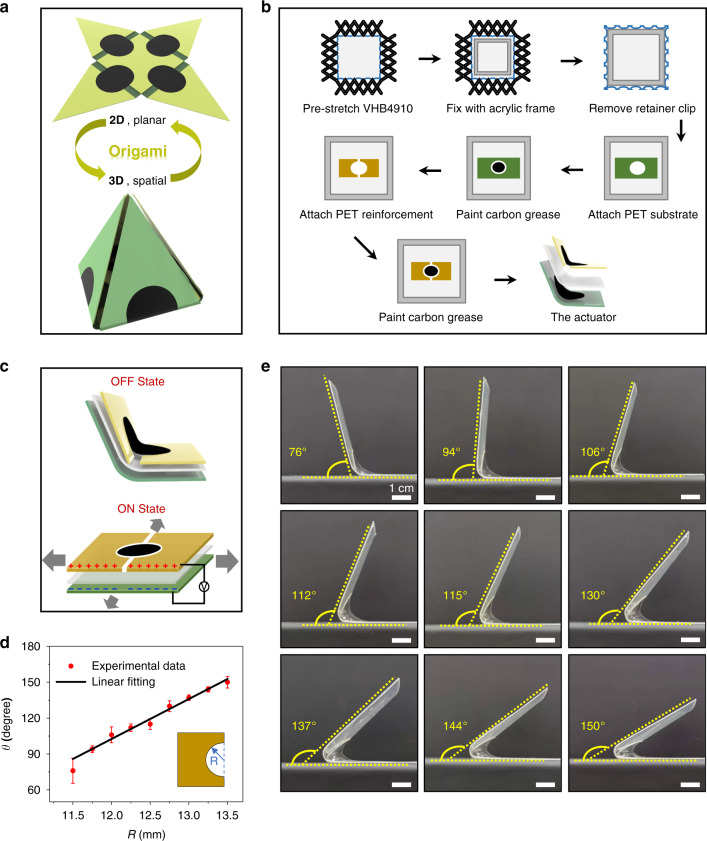
Fabrication and actuation principles of origami soft actuators with dielectric elastomers. **a** Schematic diagram of the origami-inspired soft robots. **b** Flow chart of the fabrication process for the soft actuator. After release, an actuator with a certain initial bending angle was obtained. **c** Schematic diagram of the actuation principle and the expanded layered structure of the soft actuator with a dielectric layer (VHB4910), a reinforcement layer (PET film with a thickness of 0.25 mm), and a flexible substrate (PET film with a thickness of 0.1 mm). **d** Relationship between the semicircular radius of the actuation region with the dielectric layer and the bending angle. **e** Optical images of actuators with different semicircular radii in the actuation region with different bending angles

Figure 1 shows the fabrication and actuation principles of the origami-inspired soft actuators. As shown in Fig. 1a, the planar 2D and spatial 3D structures of the origami-inspired soft robot can be easily switched by four soft actuators. The soft actuator consists of a dielectric elastomer (DE), which acts as the active deformation layer, and a laser-cut PET film, which acts as the flexible support frame. To fabricate the soft actuator, a VHB4910 DE film was first prestretched to 400% × 400% with a self-designed, precisely adjustable stretching tool (Fig. 1b and Fig. S1). The thickness of the DE film was reduced from 0.93 to 0.04 mm (Fig. S2). The stretched DE film was fixed to an acrylic frame, and a laser-cut 0.1 mm-thick PET film with a circular hole was pasted on it as a flexible frame. Then, two laser-cut 0.25 mm thick PET films with specific semicircular radii were pasted on the other side of the DE film in the same position as the reinforcement frame. To apply an actuation voltage to the DE film, conductive carbon grease was painted on both sides of the middle round area, which functioned as the actuation region. A thin wire was placed at the edge of the carbon grease electrode, and the soft actuator was actuated by a voltage source. After cutting the DE film along the outer edge of the PET film and releasing it, a soft actuator with an original saddle shape was acquired^48^.

When a voltage is applied to the actuator, charge accumulates on the flexible electrode surfaces on both sides of the film, as shown in Fig. 1c. When the accumulation reaches a certain threshold, the electrostatic force on the positive and negative electrode surfaces squeezes the middle DE film layer, causing it to expand in all directions. As the DE film expands in the actuation area, the shape of the actuator is no longer in equilibrium, resulting in bending and braking effects^49^. The braking effect is not only voltage-dependent but also related to the thickness and elastic modulus of the elastomer material. Prestretching can greatly reduce the thickness of the DE film, allowing for braking deformation with a low actuation voltage. In this work, a VHB4910 elastomer film (3 M, USA) was used as the actuator due to its high tensile rate, low elastic modulus, and low cost^50^.

As shown in Fig. 1b, e, the actuator exhibits an original bending angle in its natural state. When an external voltage is applied, the actuator tends to straighten its body, reducing the angle between the actuator and the horizontal line, which we define as the bending angle of the actuator. The original bending angle is crucial for constructing 3D origami soft robots. For example, a 90° soft actuator can be used to build square 3D origami robots, while a 120° soft actuator is the best choice for building pyramidal 3D origami robots. The original bending angle could be adjusted by the area of the actuation region. Therefore, we investigated the relationship between the bending angle and the semicircular radius of the actuation region. A set of soft actuators with two semicircular radii ranging from 11.5 to 13.5 mm spaced 6 mm apart were fabricated. The obtained soft actuators with different original bending angles are shown in Fig. 1e. The linear relationship between the bending degree and the radius is summarized in Fig. 1d. A soft actuator with a radius of 11.75 mm was bent at approximately 90°, while an actuator with a radius of 12.5 mm was bent at approximately 120°. By adjusting the radius of the actuation region, soft actuators with specific bending angles could be easily acquired. This result provides strong support for the subsequent assembly experiments with crawling robots and 3D folding assemblies with origami-inspired soft robots.

**Fig. 2:**
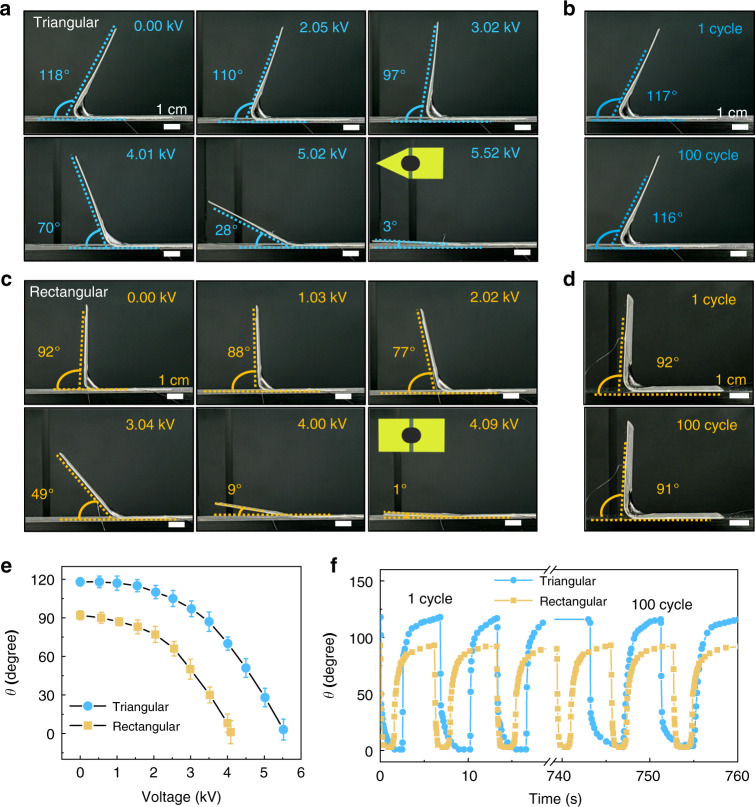
Study of triangular and rectangular soft actuators. **a** Photos of the 12.5 mm-radius triangular actuator under different actuation voltages. **b** Photos of the triangular actuator after the first and 100th actuation cycles. **c** Optical images of the 11.75 mm-radius rectangular actuator under different actuation voltages. **d** Photos of the rectangular actuator after the first and 100th actuation cycles. **e** The relationship between the bending angle and the input voltage for the triangular (blue) and rectangular (orange) actuators. **f** The stability during 100 actuation cycles for the triangular (blue) and rectangular (orange) actuators

To construct the 3D soft robots, we chose soft actuators with bending angles of 90° and 120° as examples. Figure S3 presents the planar structure design for these two soft actuators. To create a 3D pyramid soft robot, we used a 120° soft actuator with a triangular actuation side and a 12.5 mm-radius actuation region; the triangular side tended to straighten during the actuation process. Therefore, we defined this 120° soft actuator as the triangular actuator. Similarly, we used a 90° soft actuator with an 11.75 mm radius as a rectangular actuator for the 3D square soft robot. The actuation and deformation behaviors of these two soft actuators were critical for the performance of the 3D soft robots, and the electrical test results are summarized in Fig. 2. After the triangular soft actuator was connected to the power supply via thin wires, the actuation voltage was gradually increased from 0 to 5.52 kV in steps of 0.5 kV. The results show that the triangular soft actuator deformed to a horizontal state at 5.52 kV with a bending angle of only 3° (Fig. 2a). The relationship between the bending angle and the actuation voltage is shown in Fig. 2e. The triangular soft actuator also exhibited excellent cycling consistency. The cycling test results in Fig. 2b, f and Movie S1 show that the soft actuators maintained their original shape after 100 cycles. This indicates that soft actuators and 3D soft robots with DE films have a stable and reproducible performance during repeated use. For the rectangular actuator, an excellent deformation performance was also achieved, as shown in Fig. 2c–f and Movie S2. Under an actuation voltage of 4.09 kV, the rectangular soft actuator deformed to a horizontal state with a bending angle of only 1°. After 100 cycles, there was no significant difference in the shape of the actuator. We also tested the life cycle of six straight-edge DE actuators with 90° angles and found that the robots fully recovered to their initial angle within the first 500 cycles. As the number of cycles increased, the recovery characteristics of the DE actuators worsened due to the bending fatigue of the PET substrates. After 5000 cycles, these DE actuators only recovered to an angle of 60°, while they were expected to recover to 90°. In addition, the mechanical properties of both actuators were investigated by using micromechanical sensors to measure the actuator’s force at different voltages (Fig. S4). The result showed that a 3.61 mN force was generated when the triangular soft actuator was actuated by a voltage of 5.52 kV. The rectangular actuator only exhibited a force of 2.16 mN under a voltage of 4.09 kV due to the relatively small change in the bending angle.

**Fig. 3:**
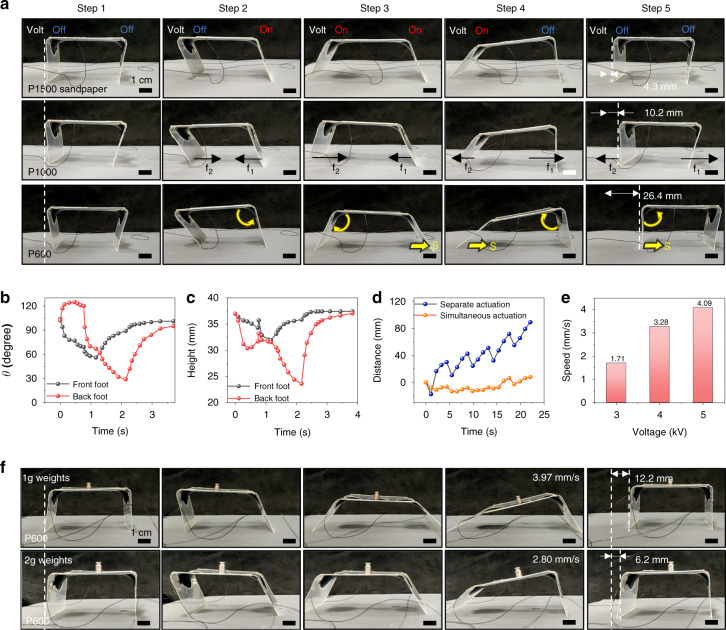
Crawling behaviors of the origami-inspired soft robot. **a** Analysis of the soft robot’s behaviors at each step of the crawling process, including the actuator’s switching state, the force direction, and the displacement direction. The soft robots walked on sandpaper with different grits, including P1500, P1000, and P600. **b** Angle variation of the soft robot’s front and rear feet with time during one motion cycle. **c** Height variation of the soft robot’s front and rear foot joints with time during one motion cycle. **d** Displacement variation with time for the soft robot under separate and simultaneous actuation. **e** Crawling speed of the soft robot under different actuation voltages. **f** Crawling behaviors of the soft robot with different loads

In nature, many animals crawl or walk by deforming and actuating their bodies or joints. The soft actuators in this work are well suited for use as an artificial muscle in a crawling robot. Therefore, we designed a 3D crawling soft robot with rectangular actuators and studied its walking behavior (Fig. 3). The soft crawling robot has a square body and rectangular bipeds at both ends. Figure S5 shows the planar structure design, which included two actuation regions with radii of 12.5 mm. The original bending angle for the two feet of this soft robot was 120°. The lengths of the feet and the main body were 40 and 60 mm, respectively. Figure 3a shows the crawling behaviors of the soft robot at each step, including the actuator’s switching state, the force direction, and the displacement direction. By actuating the front and back feet separately, the soft robot can move forward on the sandpaper. Each walking cycle can be separated into four steps (Fig. 3a and Fig. S6). In the first step, the front foot was actuated against the ground to unfold, and the soft robot tilted backward. Next, after the front foot completely unfolded, the rear foot actuated. Due to the backward shift in the center of gravity, the rear foot produced more friction force when it contacted the ground quickly, causing the soft robot to jump and crawl forward. Then, the robot’s step stabilized, and its center of gravity balanced. In the third step, the voltage on the front foot was stopped, causing it to contract, and the soft robot leaned forward, shifting its center of gravity forward again. Finally, the voltage on the rear foot was released, and the rear foot contracted and returned to its initial state because of the increased friction generated by the front foot due to the forward shift of the center of gravity. Therefore, the robot’s movement was highly dependent on the interface friction. The amount of friction generated on the surface affected the crawling displacement of the soft robot. To further investigate the effect of rough surfaces on crawling, sandpapers with various grit sizes (P1500, P1000, and P600) were used to study the crawling speed. The soft robot was actuated by a square-wave voltage with a frequency of 0.29 Hz and duty cycle of 28.6%. The bandwidth of soft actuators made from VHB4910 elastomers is usually less than 10 Hz due to the viscoelasticity of VHB elastomers. The results show that the average crawling speed of the robot on P600 sandpaper (4.09 mm/s) was nearly 30 times higher than that on P1500 sandpaper (0.15 mm/s) (Fig. 3a and Movie S3), which indicates that soft robots crawl better on rough surfaces. In addition, we measured the angle and height variations of the front and rear feet of the robot as it crawled on P600 sandpaper during one motion cycle at an input voltage of 5 kV. In this intermittent separate actuation method, the center of gravity was moved by changing the height of the robot’s feet separately; thus, the front and rear feet generated different friction forces and crawled to one side during actuation. To verify the superiority of this actuation method, the crawling robot was also actuated simultaneously at the same voltage for comparison (Fig. 3d). When the front and rear feet were actuated simultaneously, it was difficult to achieve stable motion in one direction, and the resulting movement was hesitation in one place (Fig. S7 and Movie S4). In addition to the rough sandpaper, we tested the dynamic properties of soft robots crawling on zigzag surfaces. As shown in Fig. S8, zigzag surfaces with tilt angles of 10°, 20°, and 30° were built by stacking 300 pieces of 0.9 mm-thick acrylic sheets with different zigzag serration widths. It was found that (Fig. S10 and Movie S5) the maximum speed reached 5.12 mm/s for a tilt angle of 20°, which was greater than the speeds of 3.20 and 3.88 mm/s achieved for tilt angles of 10° and 30° and greater than the maximum speed of 4.09 mm/s achieved on sandpaper at the same voltage (5 kV) and frequency (0.29 Hz). As shown in Movie S5, the speed of the robot on the zigzag surface with a tilt angle of 10° was lower due to pronounced surface slippage. On the other hand, the 30° angle of inclination formed a wide serration, which hindered bipedal actuation and reduced the speed.

The actuation voltage also affected the crawling speed of the soft robot. On P600 sandpaper, the soft robot crawled at speeds of 1.71, 3.28, and 4.09 mm/s at actuation voltages of 3, 4, and 5 kV (Movie S6). The soft robot clearly exhibited a larger deformation angle at higher voltages, with one step moving a longer distance. Soft robots can not only crawl on rough surfaces but also carry cargo. The motion status of a robot with 1 and 2 g loads is shown in Fig. 3f and Movie S7. The soft robot weighs 2.94 g and could carry up to 2.00 g while crawling, although its speed decreased from 4.09 to 2.80 mm/s. We also investigated the effect of different power on/off frequencies on the walking state of the robots. To stimulate the crawling ability of the soft robot, we used a higher actuation voltage of 5.5 kV. The results (Fig. S12) demonstrated that the walking speed first increased and then decreased as the power-off frequency increased due to the response time requirement of bipedal charging and discharging. As the frequency increased, the speed of bipedal actuation accelerated. Considering that the power-off time affects the stride angle during contraction, the robots’ pace per step decreased (Fig. S11) when the power-off time was less than the time required for bipedal contraction, reducing the crawling speed. According to Tables S1 and S2, the crawling speed of the robots in this paper was at the same level (~mm/s) as in previous reports. Table S2 compares the soft robot performance of existing DE actuator-powered robots. The clear advantage of our soft robots is their space efficiency and scalability due to the origami assembly.

**Fig. 4:**
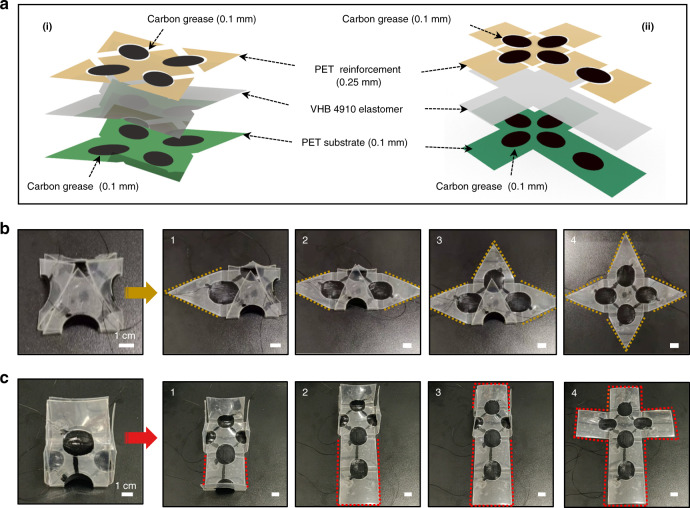
3D folding assembly of the origami-inspired soft robot. **a** Expanded multilayered diagrams of the 3D pyramid and square folding assemblies. **b** Programmable unfolding process of the pyramid-shaped soft robot. **c** Programmable unfolding process of the square-shaped soft robot

Considering the significant deformation and large actuation forces of DE-based soft actuators, they are ideal for developing complex 3D soft robots. Inspired by origami technology, these soft actuators were used to create two origami 3D soft robots with different shapes that can switch between 2D and 3D structures. Figure 4a shows expanded multilayered diagrams of the 3D pyramid- and square-shaped soft robots. The corresponding planar structure designs are presented in Figs. S13 and S14. As shown in Fig. 4b, the original shape of the origami-inspired 3D soft robot is a standard pyramid, and the pyramid-shaped 3D structure consists of four triangular actuators. Each triangular face of the pyramid can be controlled independently to open into a 2D planar structure (Movie S9). Similarly, as shown in Fig. 4c, the square-shaped 3D soft robot consists of five rectangular actuators that can be programmably actuated. Unlike the pyramid, the planar structure of the rectangular robot was not centrosymmetric, and it had a longer side that needed to withstand more gravity. The longer edges were more difficult to fold into an ideal closed-form than the shorter edges. Thus, a larger radius was needed to increase the tensile stress when folding. As shown in Fig. S14, a skeleton radius of 12.5 mm was chosen to allow the bottom of the long side to fold naturally, and the voltage required to unfold was increased from 4.1 to 5 kV, allowing a complete 3D square to be assembled (Movie S10).

**Fig. 5:**
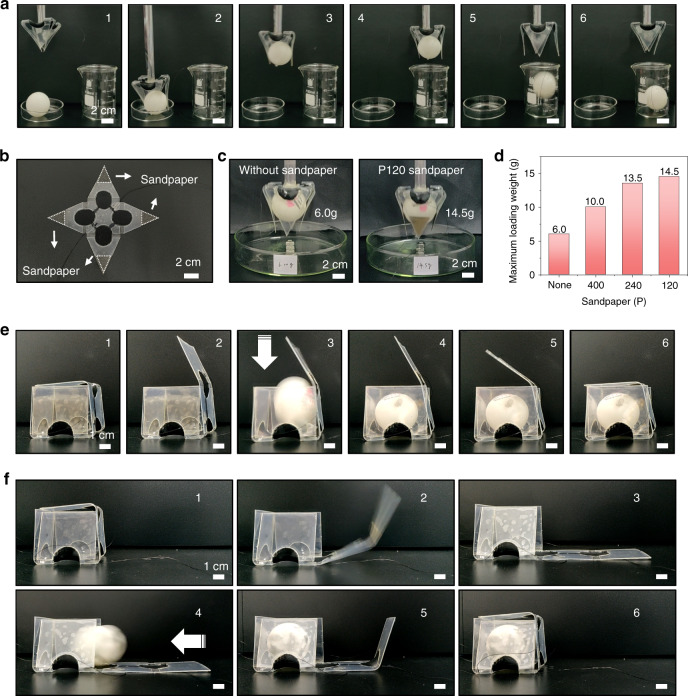
Demonstrations of the grasping and locking functions of the origami-inspired 3D soft robot acting on static and dynamic objects. **a** A pyramid-shaped soft gripper captures a static ball and transports it to a beaker. **b** Sandpaper placed on the inside of the soft gripper. **c** Sandpaper placed on the inside of the soft gripper. **d** Maximum gripping weight of the soft gripper with different grit sandpapers. **e** A square-shaped soft robot captures a falling ball and locks it in a box. **f** A square-shaped soft robot captures a rolling ball and then locks it

Soft robots for object manipulation are another important application in the field of soft robotics. The pyramid robot was transformed into a pyramid-shaped soft gripper (Fig. 5a). The back side of the pyramid-shaped gripper was attached to a rolled PET stick. The pyramid-shaped soft gripper (2.44 g) could transfer spheres (2.73 g) from a petri dish to a beaker with a finger-like grasping process. As shown in Movie S11, the soft gripper opened quickly after the actuation voltage was applied. Then, after the power was turned off, it took 2–3 s for the “finger” to completely close. The robot could pick up ping pong balls with weights up to 6.0 g. We improved the gripping ability of the soft gripper by placing rough sandpaper on the inside of the gripper. The weight of the ping pong ball was continuously increased by filling it with water, and the gripping experiment was performed in weight steps of 0.5 g. As expected (Fig. 5c, d and Movie S12), the gripping ability improved due to the roughness of the sandpaper: 10.0 g for P400, 13.5 g for P240, and 14.5 g for P120. In addition to the gripper-shaped manipulator, the square-shaped soft robot can be fabricated as a box to capture static and dynamic objects (Movie S13). The ball in Fig. 5e fell vertically, and the ball in Fig. 5f was rolled horizontally from the right side. Figure 5e illustrates the entire process of locking a falling ball. The top lid of the box was independently actuated and opened; after the ball fell into the square, the lid closed and locked the ball after stopping the actuation voltage. Figure 5f demonstrates the full process by which the square-shaped soft robot captured and locked a small ball that rolled in from the side. The long side of the rectangle was actuated independently, and it unfolded rapidly (2 s) with an applied voltage of 5 kV. After the ball rolled into the rectangular box from the right side, the long side was closed to lock the ball. The whole process took only 8 s (Movie S14). These results demonstrate the unlimited potential of origami-inspired soft robots with dielectric elastomer actuators, which have considerable advantages for multifunctional field applications in the field of soft robotics.

## Conclusion

In this work, we developed DE-based soft actuators with stable folding and unfolding functions and designed and fabricated 3D soft robots based on 3D origami folding. The soft actuator consists of a VHB4910 elastomer, which acts as the dielectric layer, and a PET film, which acts as the flexible substrate and reinforcement layer. The relationship between the semicircular radius of the actuation region and the original bending angle of the soft actuator was investigated. A triangular soft actuator with a 120° bending angle was suitable for assembling 3D pyramid-shaped soft robots, while a rectangular actuator with a 90° bending angle was used to construct a crawling soft robot and a square-shaped 3D soft robot. The stable structure of the soft actuator after 100 cycles ensured structural stability during 3D construction and durability during long-term applications of the 3D soft robots. For example, a crawling robot with rectangular soft actuators demonstrated a stable crawling ability on different grit papers and can carry cargo while walking in a specific direction. The origami-inspired 3D pyramid- and square-shaped soft robots had stable structures and could grasp and lock 3D objects. The 3D design of the assembly could also be more complex and versatile, and the DE actuator, with its advantages of a fast response time, light weight, and high durability, offers new possibilities for the development of origami soft robots. This work provides a good method for the structural and functional design of origami soft robots.

## Materials and methods

### Fabrication of the soft actuator

First, the PET flexible films, including 0.1 mm-thick substrate layers and 0.25 mm-thick reinforcement layers, were cut into specific shapes with a laser cutting machine (Mintron MC-3020) based on a pattern designed in AutoCAD. The VHB4910 elastomer (3 M, 60 mm × 60 mm) was stretched to 400% × 400% using a prestretching tool. The prestretched film was fixed using an acrylic fixation frame. Then, the PET flexible substrates and reinforcement layers with preconnected electrodes were fixed to the center of the actuator. The skeletonized area of the PET film was coated with carbon grease (AMKE G-660A). Finally, the soft actuator was obtained after it was cut and removed from the fixation frame.

### Actuation and deformation tests

The fabricated soft actuators were connected to a high-voltage DC power supply. When a certain voltage was applied, the soft actuator straightened or deformed. During the cycling test, the triangular and rectangular actuators were continuously charged and discharged to fully deform and return to their initial states at voltages of 5.5 and 4.1 kV, respectively. The cycles were repeated 100 times. All deformation processes were recorded with a camera (SONY).

### Fabrication of origami robots

The origami robots were designed with CAD software, and the PET flexible frame and reinforcement layers were made with a laser cutter. The robots were fabricated by the same process as the soft actuators, but a wire was connected to each actuation region to serve as the positive pole. The negative pole was connected to the actuation region with carbon grease (AMKE G-660A) before it was adhered to the soft substrate. Finally, a wire was used as the common negative electrode.

### Movement characterization

Two high-voltage DC power supplies were used as actuation sources to control the front and back feet of the robot. The whole crawling process was recorded using a camera (SONY).

## Supplementary information

Supporting Information

Movie S1

Movie S2

Movie S3

Movie S4

Movie S5

Movie S6

Movie S7

Movie S8

Movie S9

Movie S10

Movie S11

Movie S12

Movie S13

Movie S14
