# Supplementary material for: Origami-inspired folding assembly of dielectric elastomers for programmable soft robots
Source: Microsyst Nanoeng. 2022 Mar 31;8:37. doi: 10.1038/s41378-022-00363-5 (PMC8971403; doi:10.1038/s41378-022-00363-5)
Supplement: Supplementary file 1 — Supporting Information [file 41378_2022_363_MOESM1_ESM.docx]

Support information

**Origami-inspired folding assembly of dielectric elastomers for programmable soft robots**

*Yanhua Sun^1,2±^, Dengfeng Li^3±^, Mengge Wu^1,3±^,* *Yale Yang^1,2^, Jingyou Su^3^, Tszhung Wong^3^, Kangming Xu^2^, Ying Li^2^, Lu Li^2,*^, Xinge Yu^3,*^, Junsheng Yu^1,*^*

Email: [lli@cqwu.edu.cn](mailto:lli@cqwu.edu.cn) (L.L.), [xingeyu@cityu.edu.hk](mailto:xingeyu@cityu.edu.hk) (X.Y.), [jsyu@uestc.edu.cn](mailto:jsyu@uestc.edu.cn) (J.Y.)

These authors contributed equally: Yanhua Sun, Dengfeng Li, Mengge Wu

**1. Supplementary Video Description**

**Movie S1.** Cyclic testing of triangular actuator at an applied voltage of 5.52 kV.

**Movie S2.** Cyclic testing of rectangular actuator at an applied voltage of 4.09 kV.

**Movie S3.** Crawling performance of soft robot on the sandpapers with different grits.

**Movie S4.** Crawling behaviors of soft robot under separate and simultaneous actuations.

**Movie S5.** Crawling speed of soft robot on Zigzag surfaces with different tilt angles.

**Movie S6.** Crawling speed of soft robot under different actuation voltages.

**Movie S7.** Crawling behaviors of the soft robot with different loads.

**Movie S8.** Crawling speed of soft robot under different frequencies.

**Movie S9.** Programmable unfolding process of the pyramid-shaped soft robot.

**Movie S10.** Programmable unfolding process of the square-shaped soft robot.

**Movie S11.** Grasping and transferring of a static blob by the pyramid-shaped soft gripper.

**Movie S12.** Gripping ability test for pyramid-shaped soft grippers with different sandpapers.

**Movie S13.** A falling blob is captured and locked by using the square-shaped soft robot.

**Movie S14.** A rolling blob is captured and locked by using the square-shaped soft robot.

**2. Strain energy density calculation**

We calculated the actuator strain energy density. Since the dielectric elastomer films used in this work are generally pre-stretched by 4 times the original length, therefore, the Yeoh model that a good predictor of large deformations was chosen to describe the intrinsic structure of the films. The strain energy density equation can be defined as the Eq. (1). [1]

$W=C_{10}(\lambda_{1}^{2}+\lambda_{2}^{2}+\lambda_{3}^{2}-3)+C_{20}(\lambda_{1}^{2}+\lambda_{2}^{2}+\lambda_{3}^{2}-3)^{2}+C_{30}(\lambda_{1}^{2}+\lambda_{2}^{2}+\lambda_{3}^{2}-3)^{3}$ (1)

where λ_1_, λ_2_, λ_3_ are the elongation ratios of the material dimension in the length, width, and height directions. And C_10_, C_20_, C_30_ are the material constant, which could be determined from the fitted curve of the material tensile test. λ_1_=λ_2_=4 and λ_3_=0.043 for VHB4910 were obtained from experiments, and C_10_=6.93×10^-2^ MPa, C_20_=8.88×10^-4^ MPa, C_30_=1.67×10^-5^ MPa for VHB4910 were reported. [2] The final calculated strain energy density of the actuator is 1.67 MPa.

**3. Supplementary Tables Description**

**Table S1** The crawling speed of existing DEA-powered robots

| DEA materials | Crawling mechanism | Speed (mm/s) | Reference |
| --- | --- | --- | --- |
| SBAS | The front and rear actuators are actuated with different frequencies (1 Hz and 50 Hz). The different vibration friction drives the robot moving to one side. | 3.40 | [4] |
| PDMS | The soft robot moves forward by vibration-driven asymmetric friction. | 12.00 | [5] |
| VHB4910 | Crawling behavior is achieved by the frictional difference generated by alternate folding and unfolding of the front and rear feet. | 7.85 | This work |

**Table S2** The soft robot performance of existing DEA-powered robots

| Reference | Maximum deformation (degree) | Drive voltage (kV) | Crawling speed (mm/s) | Grasping ability | Cycle life | Deformation between 2D and 3D |
| --- | --- | --- | --- | --- | --- | --- |
| [3] | < 90 | 6 | 63.43 | / | / | No |
| [4] | 460 | 0.8 | 3.4 | / | / | No |
| [5] | < 30 | 0.45 | 12 | / | / | No |
| [6] | < 90 | 5 | 37.2 | / | / | No |
| [7] | < 90 | 6 | 11.50 | / | / | No |
| This work | 120 | 5.5 | 7.85 | 14.5g | >5000 times | Yes |

**4. Supplementary Figures Description**


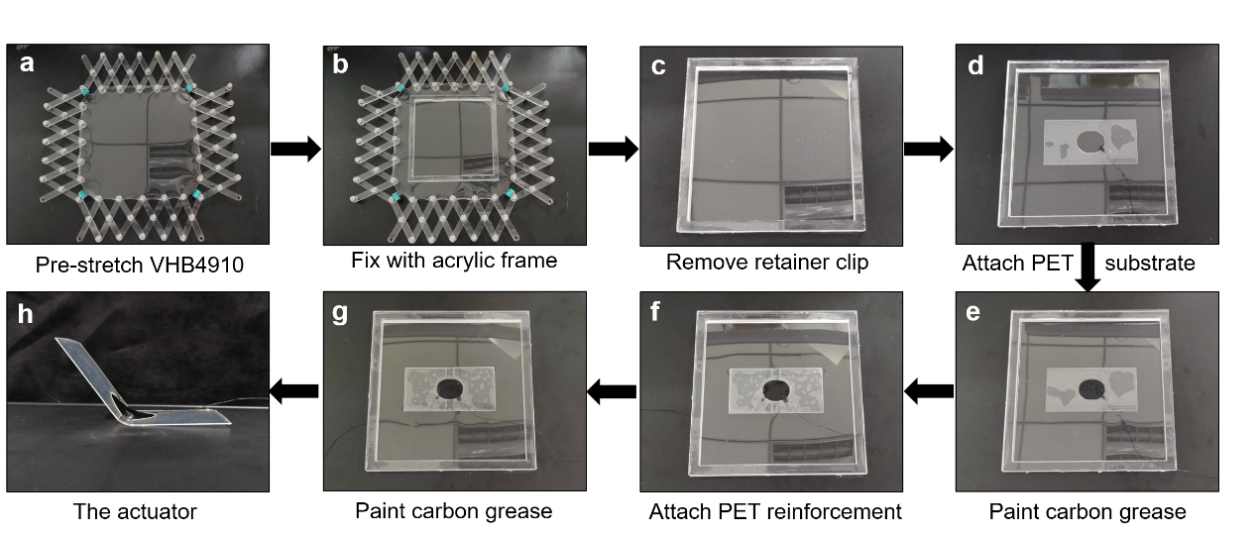


**Figure S1.** Physical flow chart of the fabrication process for the soft actuator.


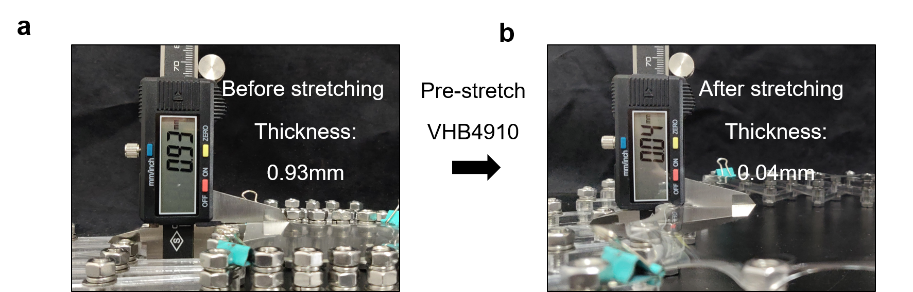


**Figure S2.** Thickness measurement of VHB4910 film before and after pre-stretching. (a) Film thickness before pre-stretching is 0.93mm; (b) Film thickness after pre-stretching is 0.04mm.
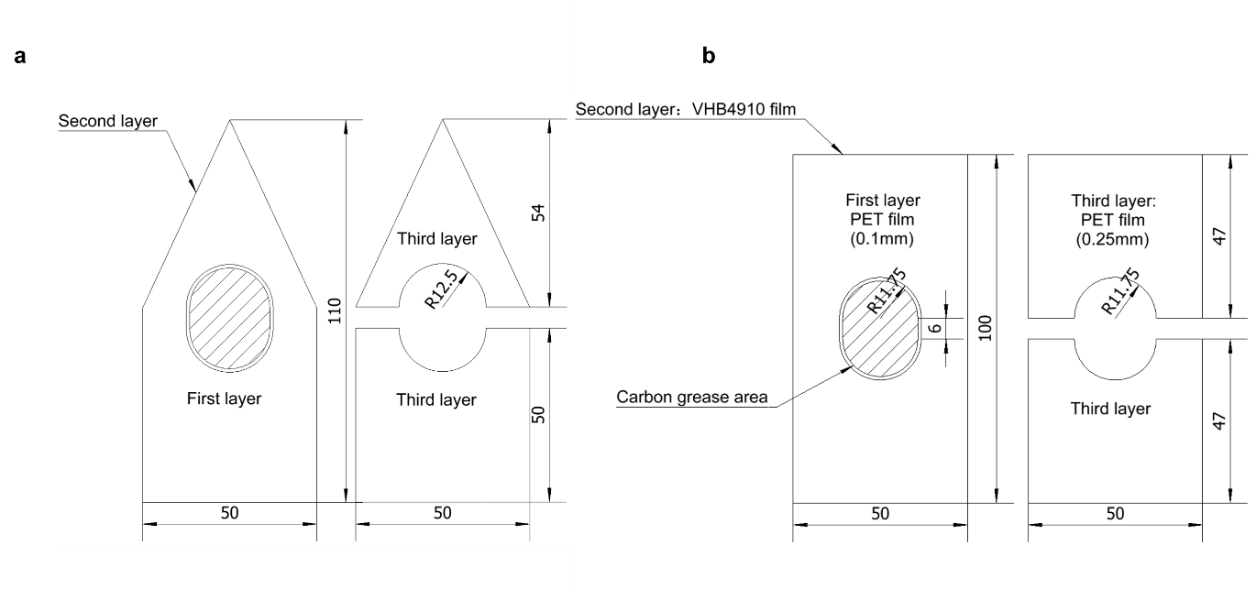


**Figure S3.** Planar structure design of soft actuators. (a) Structure of the triangular actuators with 120° original bending angle. (b) Structure of the rectangular actuators with 90° original bending angle.


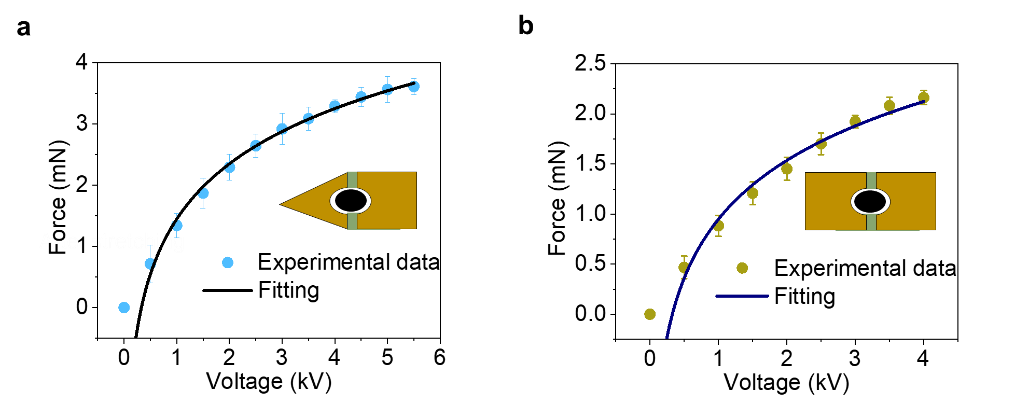


**Figure S4.** Force of the triangular and rectangular actuators at different actuation voltages. (a) Force versus voltage for the triangular actuator; (b) Force versus voltage for the rectangular actuator.


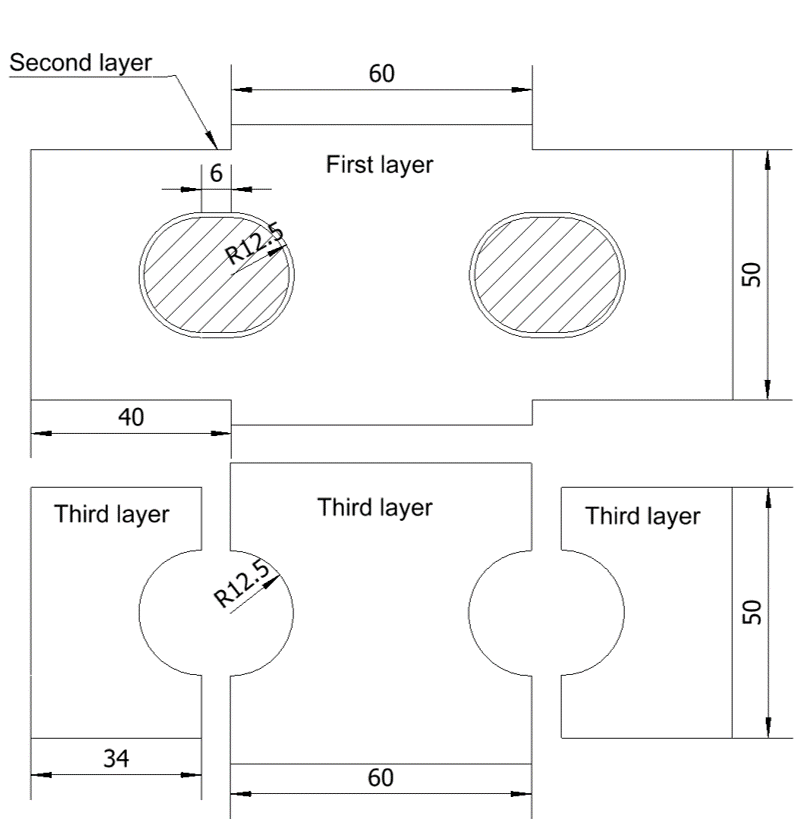


**Figure S5.** Planar structure design of crawling soft robot.


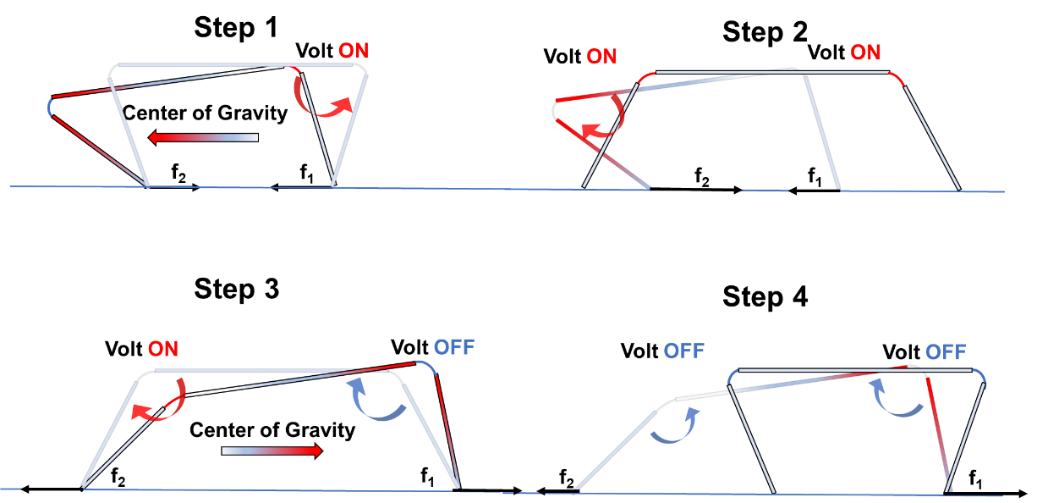


**Figure S6**. Behavior analysis of the soft robots during crawling process.


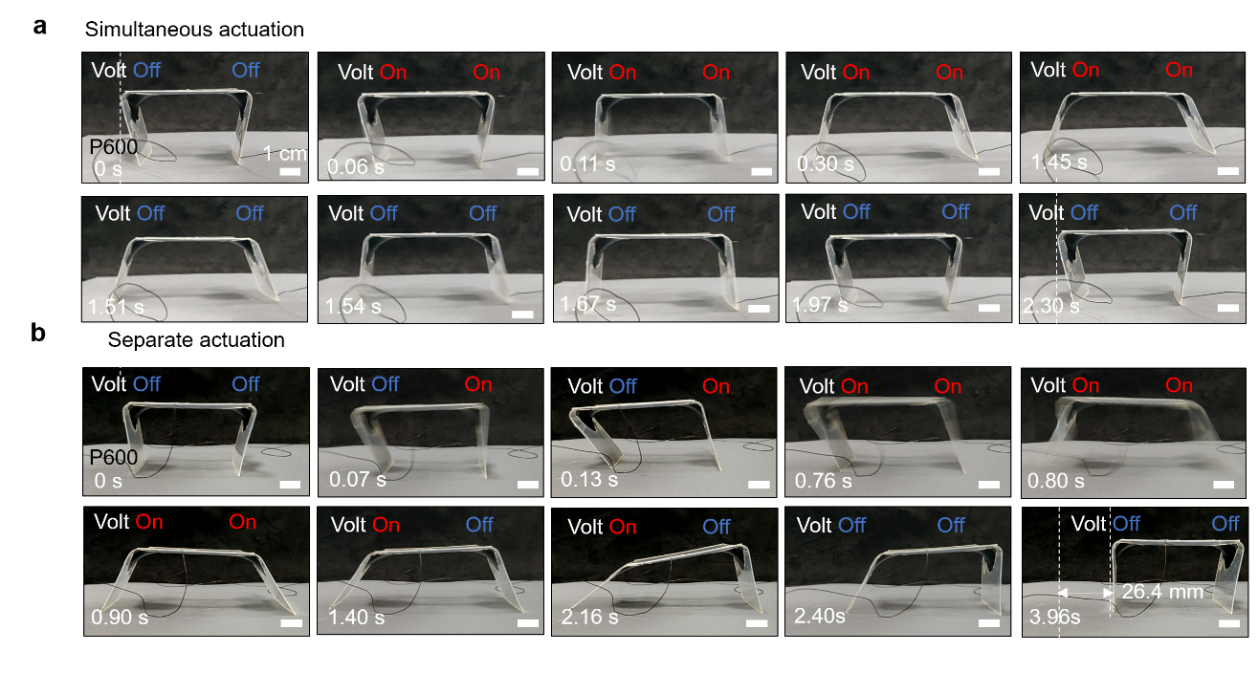


**Figure S7.** Movement performances of the soft robots under two different actuations. (a) One-cycle movement of the soft robot under simultaneous actuation; (b) One-cycle movement of the soft robot under separate actuation.


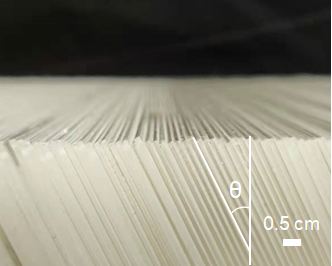


**Figure S8.** Optical image of zigzag surface built by acrylic sheets with a tilt angle of θ.


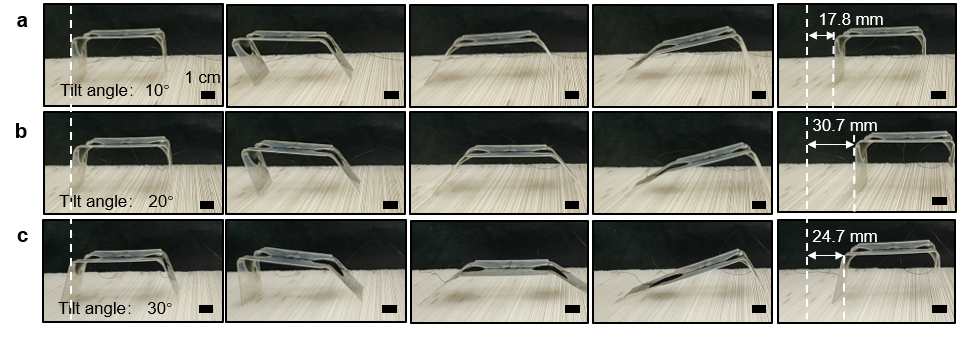


**Figure S9.** Crawling performances of soft robots on different zigzag surfaces. (a) One-cycle movement of the soft robot on a zigzag surface with a 10° tilt angle. (b) One-cycle movement of the soft robot on a zigzag surface with a 20° tilt angle. (c) One-cycle movement of the soft robot on a zigzag surface with a 30° tilt angle.


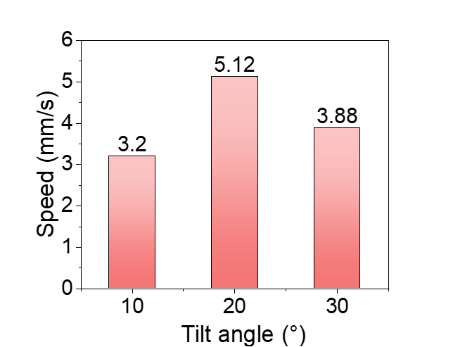


**Figure S10.** Crawling speed of soft robots on zigzag surfaces with different tilt angles.


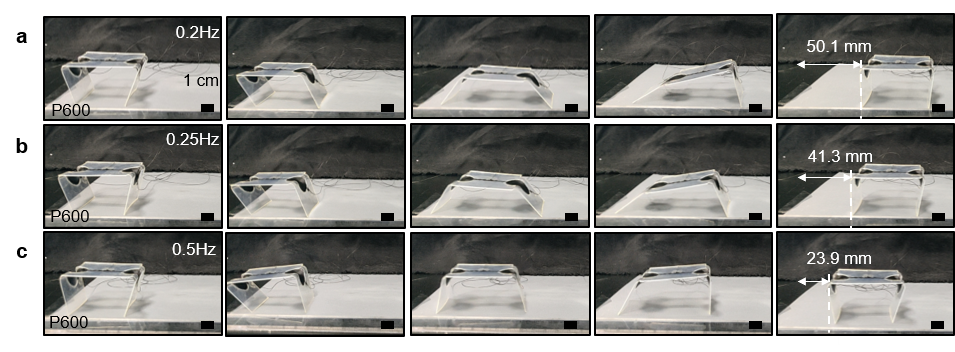


**Figure S11.** Movement performances of the soft robots under different power on/off frequencies. (a) One-cycle movement of the soft robot at 0.2Hz. (b) One-cycle movement of the soft robot at 0.25Hz. (c) One-cycle movement of the soft robot at 0.5Hz.


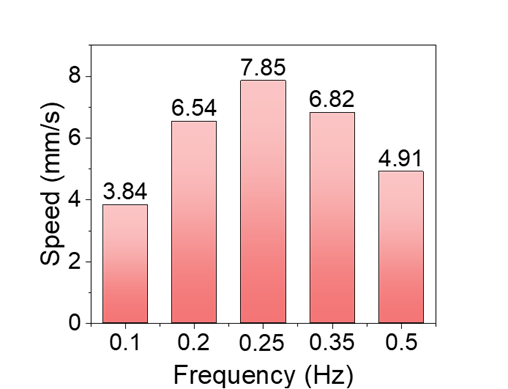


**Figure S12.** Crawling speed of soft robots on different power on/off frequencies.


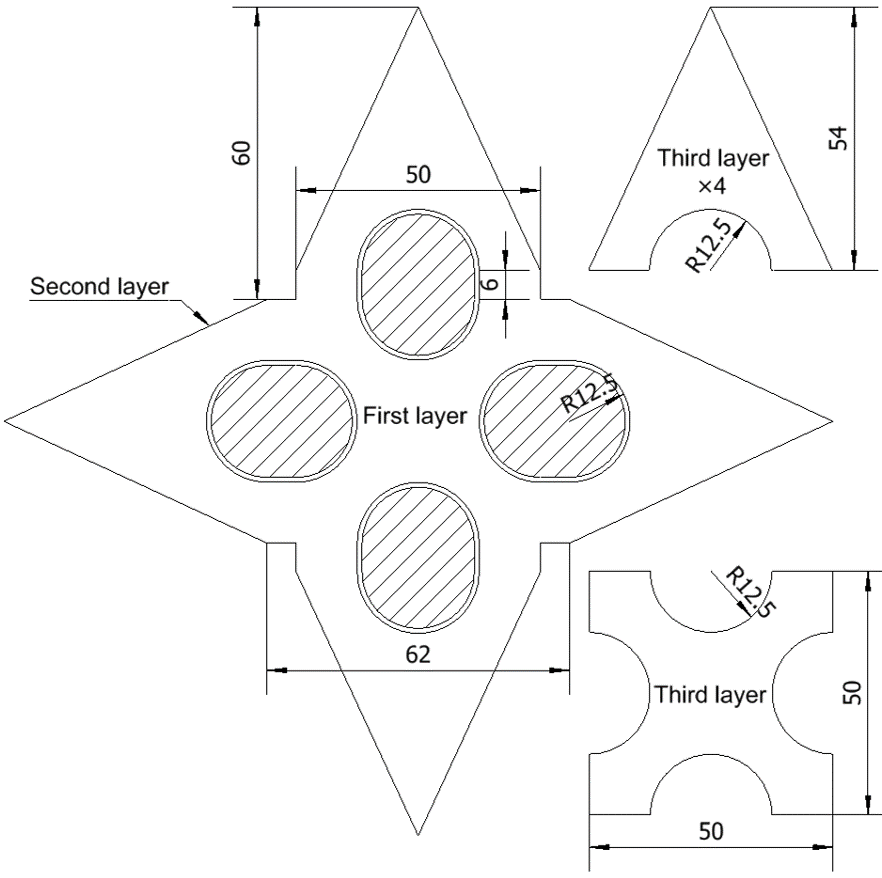


**Figure S13.** Planar structure design of the soft robot for 3D pyramid folding assembly.


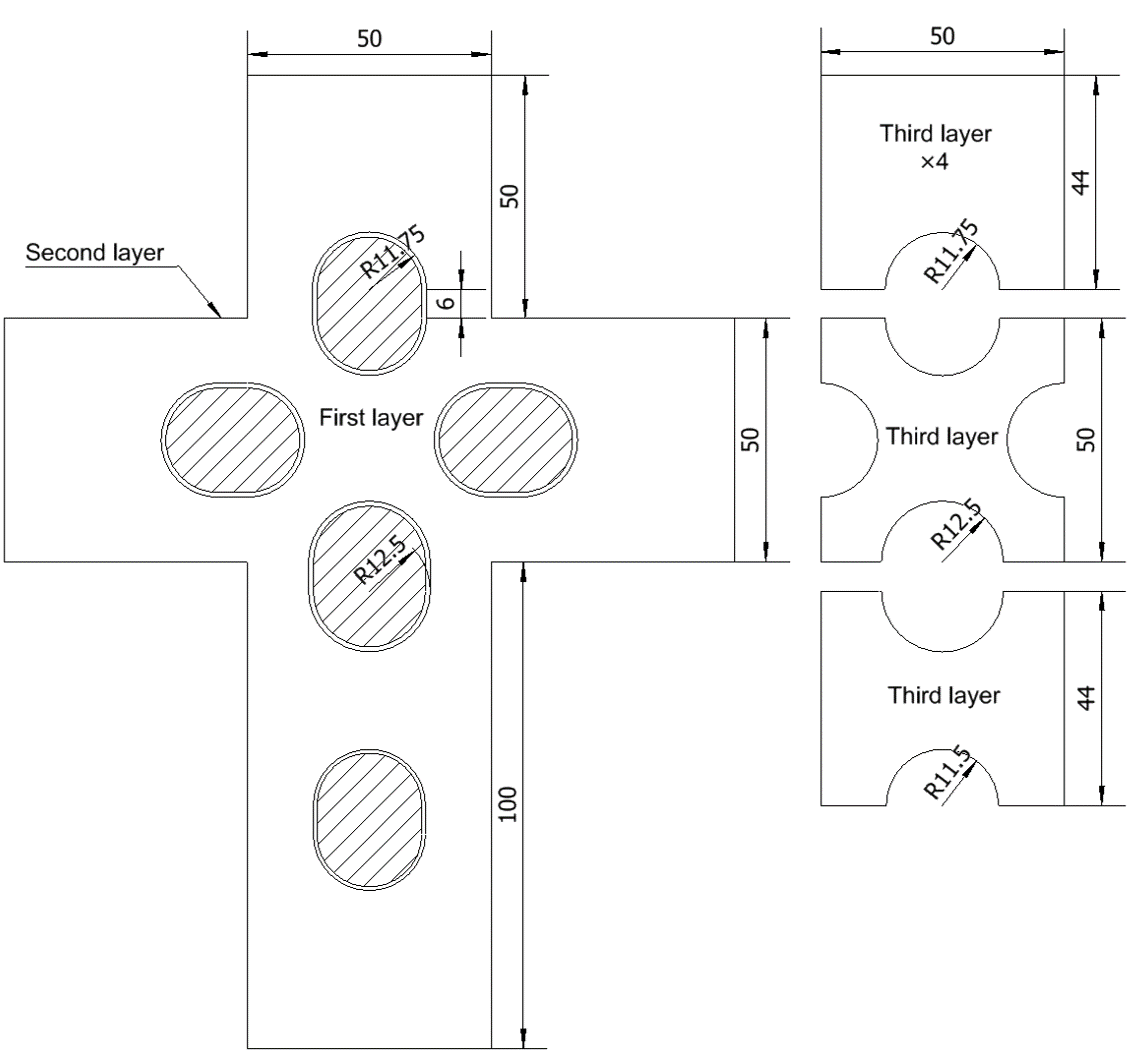


**Figure S14.** Planar structure design of the soft robot for 3D square folding assembly.

**References**

1. H. Yeoh, On the Ogden Strain-Energy Function. *Rubber Chemistry and Technology*. **70**, 175–182 (1997).
2. M. Wissler, E. Mazza, Modeling of a pre-strained circular actuator made of dielectric elastomers. *Sensors and Actuators A: Physical*. **120**, 184–192 (2005).
3. G. Gu, J. Zou, R. Zhao, X. Zhao, X. Zhu, Soft wall-climbing robots. Science Robotics. 3, eaat2874 (2018).
4. Y. Xiao, J. Mao, Y. Shan, T. Yang, Z. Chen, F. Zhou, J. He, Y. Shen, J. Zhao, T. Li, Y. Luo, Anisotropic electroactive elastomer for highly maneuverable soft robotics. Nanoscale. 12, 7514–7521 (2020).
5. X. Ji, X. Liu, V. Cacucciolo, M. Imboden, Y. Civet, A. El Haitami, S. Cantin, Y. Perriard, H. Shea, An autonomous untethered fast soft robotic insect driven by low-voltage dielectric elastomer actuators. Science Robotics. 4, eaaz6451 (2019).
6. J. Shintake, V. Cacucciolo, H. Shea, D. Floreano, Soft Biomimetic Fish Robot Made of Dielectric Elastomer Actuators. Soft Robotics. 5, 466–474 (2018).
7. X. Lu, K. Wang, T. Hu, Development of an annelid-like peristaltic crawling soft robot using dielectric elastomer actuators. Bioinspiration & Biomimetics. 15, 046012 (2020).
